# Clinical outcomes following dalbavancin administration in patients with barriers to outpatient parenteral antimicrobial therapy

**DOI:** 10.1017/ash.2022.229

**Published:** 2022-05-20

**Authors:** Jessica J. Tuan, Jehanzeb Kayani, Ann Fisher, Brian Kotansky, Louise-Marie Dembry, Rupak Datta

**Affiliations:** 1 Section of Infectious Diseases, Department of Internal Medicine, Yale University School of Medicine, New Haven, Connecticut; 2 Yale University School of Medicine, New Haven, Connecticut; 3 Hospital Epidemiology and Infection Prevention Program, Veterans Affairs Connecticut Health System, West Haven, Connecticut; 4 Department of Pharmacy, Veterans Affairs Connecticut Health System, West Haven, Connecticut

## Abstract

Between 2016 and 2021, we retrospectively identified 42 patients receiving ≥1 dose of dalbavancin for osteomyelitis, skin and soft-tissue infection, endocarditis or bacteremia, or septic arthritis. Median antibiotic duration prior to dalbavancin administration was 7 days. Within 90 days, 93% achieved clinical cure, 12% were readmitted, 12% developed hepatotoxicity, and 5% died.

Dalbavancin is an intravenous lipoglycopeptide that inhibits bacterial cell-wall linkage and is approved for the treatment of acute bacterial skin and soft-tissue infections.^1,2^ Accumulating literature supports the off-label use of dalbavancin to treat a variety of infections due to *Staphylococcus* spp, *Streptococcus* spp, and *Enterococcus* spp as well as anaerobic organisms.^3–6^ Due to its prolonged half-life, it may confer benefits in the management of invasive infections in patients with barriers to outpatient parenteral antimicrobial therapy (OPAT).^7^ We evaluated outcomes following dalbavancin administration to improve quality of care at a single institution.

## Methods

We identified patients at the Veterans Affairs Connecticut Healthcare System who received at least 1 dose of dalbavancin between January 31, 2016 and January 31, 2021. Electronic medical records were reviewed by infectious disease physicians (J.T., A.F., and R.D.) for demographics, comorbidities, microbiological data, antibiotic duration prior to dalbavancin administration, hospitalization characteristics, type of infection, and indication for dalbavancin. Indication for dalbavancin was divided into 4 categories: ineligibility for OPAT, pharmacologic factors, ineligibility for peripherally inserted central catheter (PICC), or active substance use disorder. Ineligibility for OPAT included factors such as inability to administer antibiotics at home, unstable housing, patient preference to avoid prolonged course of intravenous antibiotics, out-of-state residence, or discharge from the hospital against medical advice. Pharmacologic factors included allergy or adverse effects with other antibiotic classes, desire to avoid potential drug reactions with other antibiotic classes, increased susceptibility to dalbavancin, improved dosing regimen with dalbavancin, and therapeutic failure with other antibiotic classes. Ineligibility for PICC included patient preference to avoid PICC, prior adverse events with PICC line, or inability by patient to use PICC line. Active substance use disorder was defined as the use of recreational opiates, cocaine, or methamphetamine in any form documented during the evaluation period.

All patients were evaluated for the following outcomes: hepatotoxicity (defined as an aspartate aminotransferase or alanine aminotransferase elevation ≥3 times the upper limit of normal, or alkaline phosphatase elevation ≥2 times the upper limit of normal, or a ≥3-fold increase in total bilirubin within 14 days of dalbavancin administration); clinical cure (defined as documented resolution of infection by a physician within 90 days of dalbavancin administration); all-cause readmission (within 90 days of dalbavancin administration); and all-cause mortality (within 90 days of dalbavancin administration). Among patients who were readmitted or died, we further determined whether readmissions or deaths were associated with infection. This study was deemed quality improvement by the Veterans Affairs Health Services Research and Development Service, which waived the need for further institutional review board approval.

## Results

We identified 42 patients who received dalbavancin during the study period. Descriptive characteristics are shown in Table 1. The median hospital duration among patients receiving dalbavancin was 8 days (range, 1–32), and 4 patients (10%) required critical care. The median duration of antibiotic therapy prior to the first dose of dalbavancin was 7 days (range, 1–42) and varied by infection: septic arthritis (median, 1 day; range, 1–1); skin and soft-tissue infection (median, 5 days; range, 1–12); endocarditis or bacteremia (median, 8 days; range, 2–30); and osteomyelitis (median, 9 days; range, 1–42). All patients received dalbavancin 1,500 mg prior to hospital discharge. Of the 25 patients scheduled for a second dose of dalbavancin 1,500 mg, 23 patients (92%) received their second dose a median of 7 days (range, 4–11) thereafter. The most common infection managed with dalbavancin was osteomyelitis (n = 21, 50%); 22 infections (52%) were primarily due to *Staphylococcus* spp and 13 (31%) were polymicrobial. The latter included a mix of aerobic and/or anaerobic gram-positive organisms (eg, *Staphylococcus* spp, *Streptococcus* spp, and *Finegoldia magna*) and gram-negative organisms (eg, *Pseudomonas* spp, *Serratia marcescens*, and *Morganella morganii*). Also, 21 patients (50%) received dalbavancin due to ineligibility for OPAT. Among 13 patients with polymicrobial infections, 10 received concomitant oral antibiotics including ciprofloxacin, metronidazole, and trimethoprim/sulfamethoxazole.

**Table 1.** Characteristics of Patients Receiving Dalbavancin Between January 31, 2016 and January 31, 2021

Characteristic	No. (%)
**Age**	
30–44 y	3 (7)
45–65 y	17 (40)
66–75 y	16 (38)
≥76 y	6 (14)
Sex, male	42 (100)
**Comorbidities**	
Diabetes mellitus	23 (55)
Liver disease	13 (31)
Active substance use disorder	6 (14)
**Type of infection**	
Osteomyelitis	21 (50)
Skin and soft-tissue infection	10 (24)
Endocarditis or bacteremia	9 (21)
Septic arthritis	2 (5)
**Inpatient consultations**	
Infectious diseases	42 (100)
Podiatry	19 (45)
Orthopedic surgery	4 (10)
Interventional radiology	3 (7)
Cardiothoracic surgery	2 (5)
Vascular surgery	2 (5)
General surgery	2 (5)
Plastic surgery	1 (2)
**Microbiologic culture data**	
*Staphylococcus* spp	22 (52)
Polymicrobial	13 (31)
*Corynebacterium* spp	10 (24)
*Streptococcus* spp	5 (12)
Empiric	4 (10)
**Indication for Dalbavancin**	
Ineligible for outpatient parenteral antibiotic therapy	21 (50)
Pharmacologic factors	10 (24)
Ineligible for peripherally inserted central catheter	6 (14)
Active substance use disorder	6 (14)
**Outcomes**	
Clinical cure	39 (93)
Readmission	5 (12)
Hepatotoxicity	5 (12)
Mortality	2 (5)

During the 90 days following receipt of dalbavancin, no patients were lost to follow-up. Overall, 39 (93%) of 42 patients achieved clinical cure within 90 days of receipt of dalbavancin: 9 of 9 with endocarditis or bacteremia; 1 of 2 with septic arthritis; 20 of 21 with osteomyelitis; and 9 of 10 with skin or soft-tissue infection. Of the 3 patients who did not achieve clinical cure, 1 had a polymicrobial infection. Of 42 patients, 5 (12%) were readmitted, including 4 patients with osteomyelitis and 1 patient with septic arthritis. In addition, 3 readmissions were associated with infection, all of which were managed with repeat surgery when applicable. All-cause mortality was 2 (5%) of 42, and 1 death was associated with osteomyelitis. Hepatotoxicity occurred in 5 (12%) of 42 patients, none of whom developed secondary complications of drug-induced hepatitis.

## Discussion

The management of invasive bacterial infections in patients with barriers to OPAT is challenging. Prior studies in this population have largely focused on persons who inject drugs.^4,5^ We conducted a 5-year, single-center analysis of all patients with barriers to OPAT, such as home stability, patient preference, pharmacologic factors, and inability to use a PICC, in addition to active substance use disorder. Our findings suggest that dalbavancin offers therapeutic benefit in this heterogenous group. Combined with prior literature, our data support the use of dalbavancin as secondary therapy for osteomyelitis, endocarditis, and septic arthritis in patients with barriers to OPAT.^8^

Our study of patients receiving care within the Veterans Health Administration facilitated comprehensive follow-up of patients. However, this setting may reduce the external validity of our findings. The single-center design, small sample size, and lack of representation from women further limit generalization of this work. In addition, it is difficult to distinguish the clinical benefit of dalbavancin from the effectiveness of prior or concomitant antibiotic therapy.

Nevertheless, this work provides evidence that dalbavancin may be considered in patients with invasive bacterial infections for whom daily intravenous therapy may be nonviable. Future studies are needed from larger cohorts, perhaps using administrative data across healthcare systems such as the Veterans’ Health Administration, to confirm these findings.
